# Crystal structure of {*N* ^1^,*N* ^3^-bis­[(1-*tert*-butyl-1*H*-1,2,3-triazol-4-yl)methyl­idene]-2,2-di­methyl­propane-1,3-di­amine}­bis­(thio­cyanato)­iron(II)

**DOI:** 10.1107/S2056989021004412

**Published:** 2021-04-30

**Authors:** Kateryna Znovjyak, Maksym Seredyuk, Sergey O. Malinkin, Iryna O. Golenya, Vladimir M. Amirkhanov, Sergiu Shova, Nurullo U. Mulloev

**Affiliations:** a Department of Chemistry, Taras Shevchenko National University of Kyiv, Volodymyrska Street 64, Kyiv, 01601, Ukraine; b Department of Inorganic Polymers, "Petru Poni" Institute of Macromolecular, Chemistry, Romanian Academy of Science, Aleea Grigore Ghica Voda 41-A, Iasi, 700487, Romania; c The Faculty of Physics, Tajik National University, Rudaki Avenue 17, Dushanbe, 734025, Tajikistan

**Keywords:** iron(II) complex, thio­cyanate complex, high-spin state, trigonal distortion, magnetism, energy frameworks, crystal structure

## Abstract

The unit cell of the title compound, [Fe^II^(NCS)_2_(C_19_H_32_N_8_)], consists of two charge-neutral complex mol­ecules. In the complex mol­ecule, the tetra­dentate ligand *N* ^1^ *,N* ^3^-bis­[(1-*tert*-butyl-1*H*-1,2,3-triazol-4-yl)methyl­ene]-2,2-di­methyl­propane-1,3-di­amine coordinates to the Fe^II^ ion through the N atoms of the 1,2,3-triazole and aldimine groups. Two thio­cyanate anions, also coordinated through their N atoms, complete the coordination sphere of the central Fe ion. In the crystal, neighbouring mol­ecules are linked through weak C—H⋯C/S/N inter­actions into a three-dimensional network. The inter­mol­ecular contacts were qu­anti­fied using Hirshfeld surface analysis and two-dimensional fingerprint plots, revealing the relative contributions of the contacts to the crystal packing to be H⋯H 50.8%, H⋯C/C⋯H 14.3%, H⋯S/S⋯H 20.5% and H⋯N/N⋯H 12.1%. The average Fe—N bond distance is 2.170 Å, indicating the high-spin state of the Fe^II^ ion, which does not change upon cooling, as demonstrated by low-temperature magnetic susceptibility measurements. DFT calculations of energy frameworks at the B3LYP/6–31 G(d,p) theory level were performed to account for the inter­actions involved in the crystal structure.

## Abstract 2

The title charge-neutral complex shows a *cis*-arrangement of the thio­cyanate anions, with a severely distorted coordination octa­hedron. The three-dimensional supra­molecular architecture of the lattice is formed by weak C—H⋯C/S/N hydrogen bonds.

## Chemical context   

An inter­esting class of coordination compounds exhibiting spin-state switching between low- and high-spin states is represented by Fe^II^ complexes based on Schiff bases derived from N-substituted 1,2,3-triazole aldehydes (Hagiwara *et al.*, 2014 ▸, 2016 ▸, 2020 ▸; Hora & Hagiwara, 2017 ▸). In all of the charge-neutral mononuclear complexes of this kind described so far, the thio­cyanate anions occupy the axial position in the coord­ination sphere and thus are in a *trans*-configuration (Hagiwara & Okada, 2016 ▸; Hagiwara *et al.*, 2017 ▸).
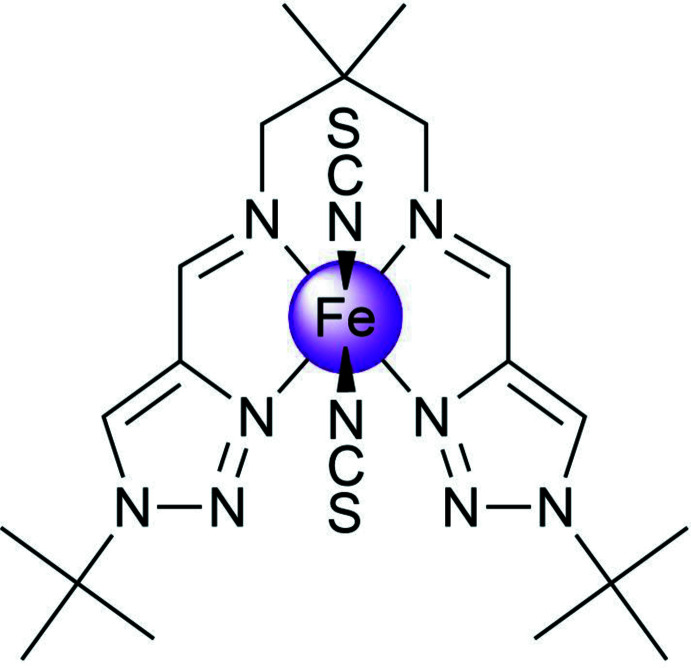


Having inter­est in functional 3*d* metal complexes formed by polydentate ligands (Seredyuk *et al.*, 2006 ▸, 2007 ▸, 2011 ▸, 2012 ▸, 2015 ▸, 2016 ▸; Valverde-Muñoz *et al.*, 2020 ▸), we report here a continuation of our ongoing exploration of new Fe^II^ *cis*-complexes with thio­cyanate anions and tetra­dentate ligands *N* ^1^,*N* ^3^-bis­[(1-*R*-1*H*-1,2,3-triazol-4-yl)methyl­ene]-2,2-di­methyl­propane-1,3-di­amine, and report below structural and magnetic investigations of a new complex with *R* = *tert*-butyl.

## Structural commentary   

**Figure 1:**
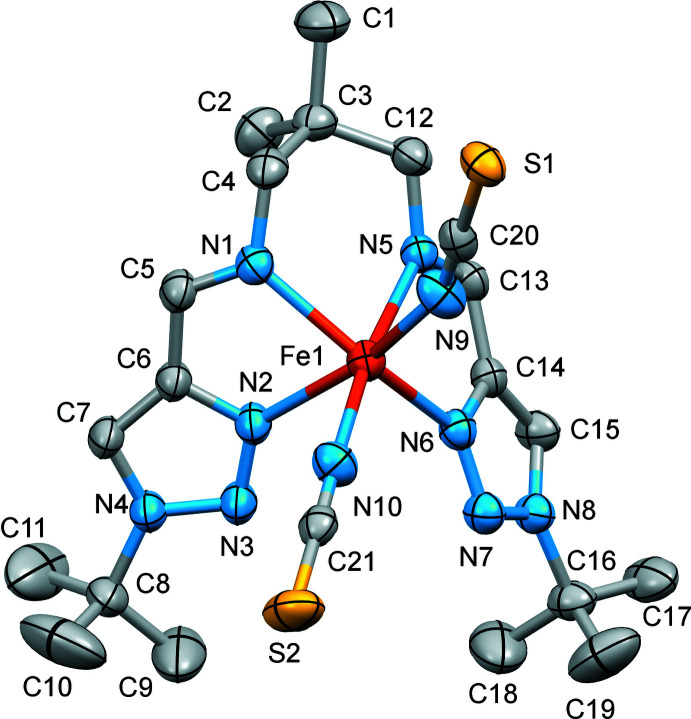
The mol­ecular structure of the title compound with displacement ellipsoids drawn at the 50% probability level. H atoms have been omitted for clarity.

The Fe^II^ ion of the title complex has a distorted trigonal–prismatic N_6_ coordination environment formed by the four N atoms of the tetra­dentate Schiff-base ligand and the two NCS^−^ counter-ions (Fig. 1 ▸). The average bond length, <Fe—N> = 2.170 (4) Å, is typical for high-spin complexes with an [FeN_6_] chromophore (Gütlich & Goodwin, 2004 ▸). The N—Fe—N′ angle between the *cis*-aligned thio­cyanate N atoms is 91.91 (8)°. The average trigonal distortion parameters, *Σ* = Σ_1_^12^(|90 – *φ_i_*|), where *φ*_i_ is the angle N—Fe—N′ (Drew *et al.*, 1995 ▸), *Θ* = Σ_1_^24^(|60 – *θ_i_*|), where *θ_i_* is the angle generated by superposition of two opposite faces of an octa­hedron (Chang *et al.*, 1990 ▸) are 127.8 and 438.2°, respectively. The values reveal a great deviation of the coordination environment from an ideal octa­hedron (where *Σ* = *Θ* = 0), and are significantly larger than those of similar [FeN_6_] high-spin *trans*-complexes (Hagiwara *et al.*, 2017 ▸). With the aid of continuous shape measurements (CShM), the closest shape of a coordination polyhedron and its distortion can be determined numerically (Kershaw Cook *et al.*, 2015 ▸). The calculated CShM value relative to ideal *O*_h_ symmetry is 3.829, while it is 6.709 relative to the ideal *D*_3h_ trigonal–prismatic symmetry. Hence, the polyhedron is closer to the former geometry, but is still appreciably distorted, as indicated by the calculated value (for an ideal polyhedron CShM = 0). The volume of the [FeN_6_] coordination polyhedron is 12.60 Å^3^.

## Supra­molecular features   

**Figure 2:**
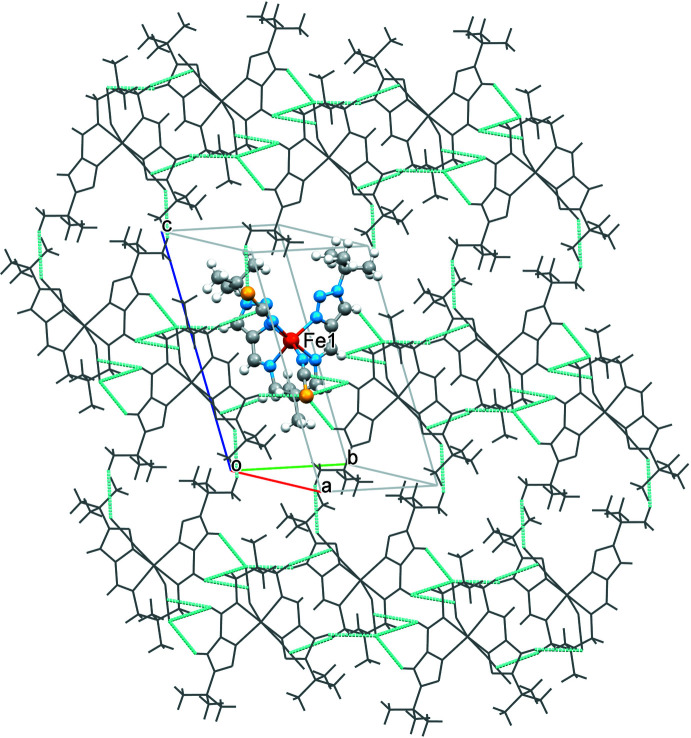
The packing of mol­ecules into the three-dimensional network held together by weak C—H⋯C/S bonding (dashed cyan lines).

In the lattice, neighbouring complex mol­ecules form a three-dimensional supra­molecular network (Fig. 2 ▸) through the weak C—H⋯*X* hydrogen bonds (Table 1 ▸). No strong hydrogen bonding or stacking inter­actions are observed between the complex mol­ecules in the crystal lattice.

## Hirshfeld surface and 2D fingerprint plots   

**Figure 3:**
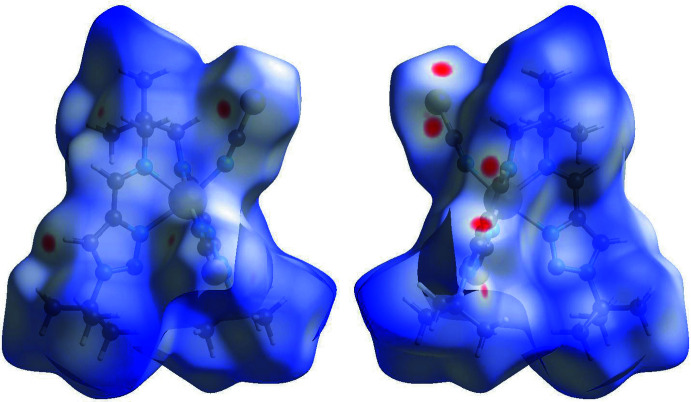
Two projections of *d*_norm_ mapped on Hirshfeld surfaces, showing the inter­molecular inter­actions within the mol­ecule. Red areas represent regions where contacts are shorter than the sum of the van der Waals radii, blue areas represent regions where contacts are larger than the sum of van der Waals radii, and white areas are regions where contacts are close to the sum of van der Waals radii.

**Figure 4:**
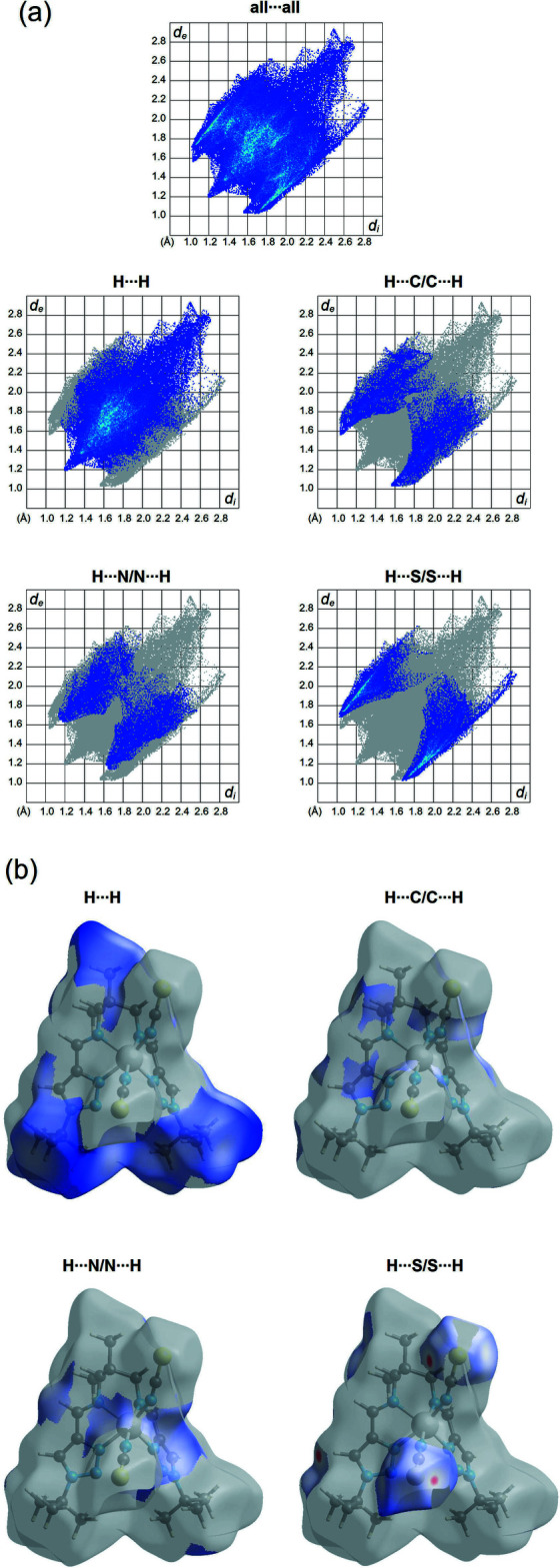
(*a*) The overall two-dimensional fingerprint plot and those decomposed into specified inter­actions. (*b*) Hirshfeld surface representations with the function *d*_norm_ plotted onto the surface for the different inter­actions.

Hirshfeld surface analysis was performed and the associated two-dimensional fingerprint plots were generated using *Crystal Explorer* (Turner *et al.*, 2017 ▸), with a standard resolution of the three-dimensional *d*_norm_ surfaces plotted over a fixed colour scale of −0.1141 (red) to 1.9978 (blue) a.u. The pale-red spots symbolize short contacts and negative *d*_norm_ values on the surface correspond to the inter­actions described above. The overall two-dimensional fingerprint plot is illus­trated in Fig. 3 ▸. The Hirshfeld surfaces mapped over *d*_norm_ are shown for the H⋯H, H⋯C/C⋯H, H⋯S/S⋯H, and H⋯N/N⋯H contacts, and the two-dimensional fingerprint plots are presented in Fig. 4 ▸, associated with their relative contributions to the Hirshfeld surface. At 50.8%, the largest contribution to the overall crystal packing is from H⋯H inter­actions, which are located in the middle region of the fingerprint plot. H⋯C/C⋯H contacts contribute 14.3%, and the H⋯S/S⋯H contacts contribute 20.5% to the Hirshfeld surface, both resulting in a pair of characteristic wings. The H⋯N/N⋯H contacts, represented by a pair of sharp spikes in the fingerprint plot, make a 12.1% contribution to the Hirshfeld surface.

## Energy frameworks   

**Figure 5:**
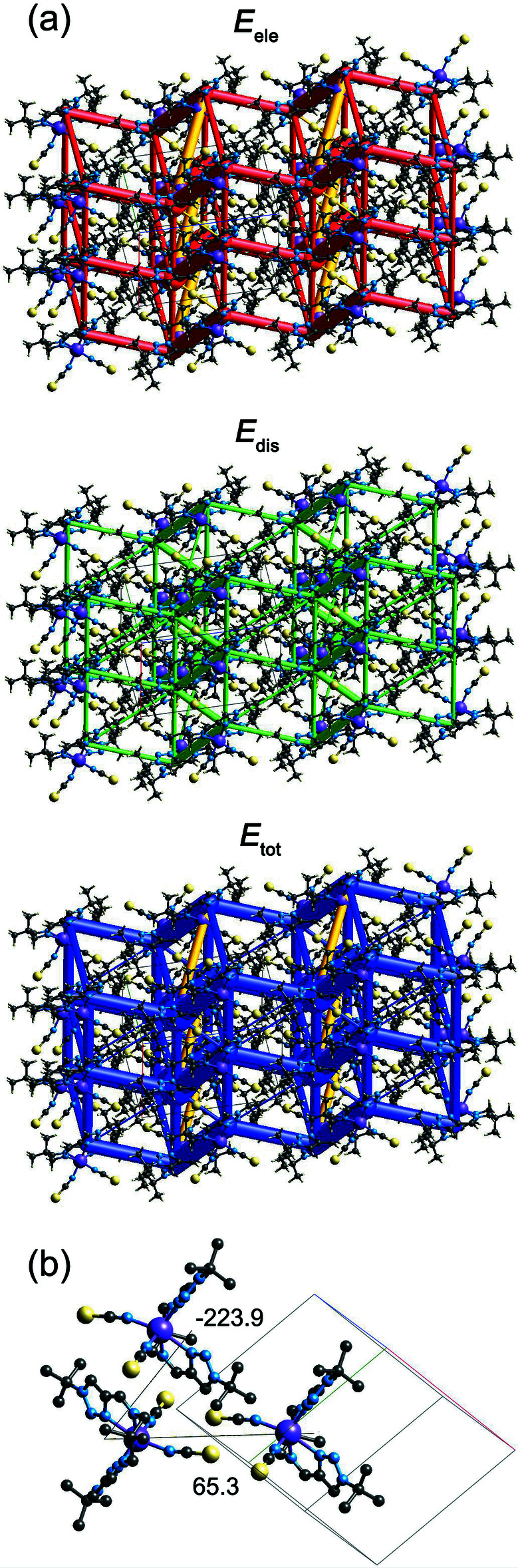
(*a*) The calculated energy frameworks, showing the electrostatic potential forces (*E*_ele_), the dispersion forces (*E*_dis_) and the total energy diagrams (*E*_tot_). Yellow coloured tubes correspond to the repulsive inter­actions; (*b*) the strongest repulsive and attractive inter­actions between neighbouring complex mol­ecules.

The energy frameworks, calculated using the wave function at the B3LYP/6-3G(d,p) level of theory for the title compound, including the electrostatic potential forces (*E*_ele_), the dispersion forces (*E*_dis_) and the total energy diagrams (*E*_tot_), are shown in Fig. 5 ▸ *a*. The cylindrical radii, adjusted to the same scale factor of 80, are proportional to the relative strength of the corresponding energies (Turner *et al.*, 2017 ▸; Tan *et al.*, 2019 ▸). It can be seen that the major contribution to the inter­molecular inter­actions is from Coulomb forces (*E*_ele_), reflecting dipole–dipole inter­actions of the asymmetric complex *cis*-mol­ecules in the lattice. According to the calculations, the most repulsive inter­action is due to the anion-to-anion alignment of neighbouring complex mol­ecules (*E*_tot_ = 65.3 kJ mol^−1^) while the ligand-to-anion alignment gives the most attractive one (*E*_tot_ = −223.9 kJ mol^−1^) (Fig. 5 ▸ *b*). The colour-coded inter­action mappings within a radius of 3.8 Å of a central reference mol­ecule for the title compound together with full details of the various contributions to the total energy (*E*_tot_) are given in the Supporting Information.

## Magnetic properties   

**Figure 6:**
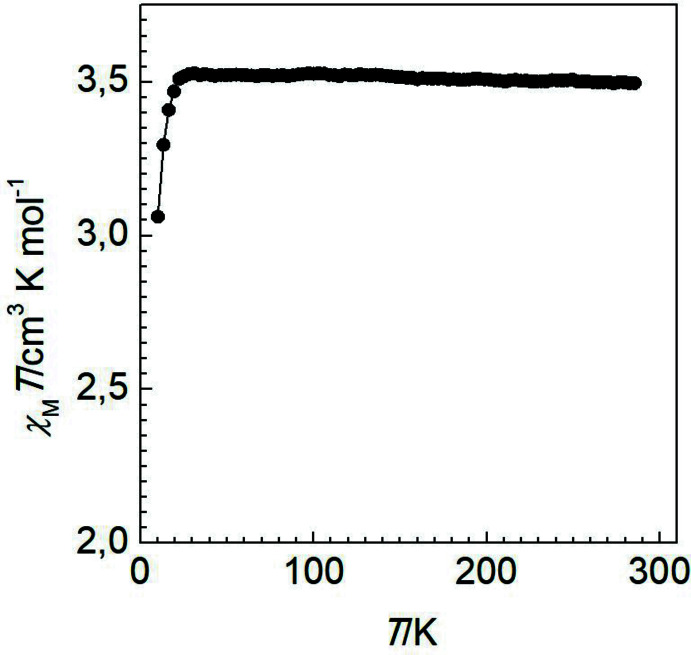
*χ*_M_ *T versus T* plot for the title compound.

Variable-temperature magnetic susceptibility measurements were performed on single crystals (10 mg) of the title compound using a Quantum Design MPMS2 superconducting quantum inter­ference device (SQUID) susceptometer operating at 1 T. Experimental susceptibilities were corrected for the diamagnetism of the holder (gelatine capsule) and of the constituent atoms by the application of Pascal’s constants. The magnetic behaviour of the compound is shown in Fig. 6 ▸ in the form of *χ*_M_ *T* versus *T* (*χ*_M_ is the molar magnetic susceptibility and *T* is the temperature). At 300 K, the *χ*_M_ *T* value is close to 3.51 cm^3^ K mol^−1^, and on cooling the value remains constant down to 30 K. The decrease of *χ*_M_ *T* below 30 K is attributed to the zero-field splitting of the high-spin (*S* = 2) Fe^II^ centres (Kahn, 1993 ▸), which corroborates with the observed long average Fe—N bond length and the large geometric distortion of the coordination polyhedron of the central Fe^II^ ion.

## Database survey   

A search of the Cambridge Structural Database (CSD, Version 5.42, last update February 2021; Groom *et al.*, 2016 ▸) reveals five similar Fe^II^ thio­cyanate complexes, derivatives of a 1,3-di­amine and *N*-substituted 1,2,3-triazole aldehydes: DURXEV, ADAQUU, ADAREF and solvatomorphs ADAROP and ADARUV (Hagiwara *et al.*, 2017 ▸, Hagiwara & Okada, 2016 ▸). These complexes show hysteretic spin crossover with variation of the Fe—N distances in the range 1.931–1.959 Å for the low-spin state and 2.154–2.169 Å for the high-spin state of the Fe^II^ ions. The reported pseudo-trigonal–prismatic complexes with an [FeN_6_] chromophore are formed by structurally hindered rigid hexa­dentate ligands favouring trigonal geometry of the central Fe^II^ ion: CABLOH (Voloshin *et al.*, 2001 ▸), BUNSAF (El Hajj *et al.*, 2009 ▸), OWIHAE (Seredyuk *et al.*, 2011 ▸), OTANOO (Stock *et al.*, 2016 ▸). The recently reported by us *cis*-complexes CUWQAP and IQEFAO have similar strongly distorted coordination environment of the central Fe^II^ ion (Znovjyak *et al.*, 2020 ▸, 2021 ▸). Table 2 ▸ collates the distortion parameters *Σ*, *Θ* and CShM for the pseudo-trigonal-prismatic complexes mentioned above.

## Synthesis and crystallization   

The synthesis of the title compound is identical to that reported by us recently for similar thio­cyanate complexes (Znovjyak *et al.*, 2020 ▸, 2021 ▸). The ligand of the title compound was obtained *in situ* by condensation of 2,2-dimethyl-1,3-propanedi­amine (24 µL, 0.20 mmol) with 1-*tert*-butyl-1*H*-1,2,3-triazole-4-carbaldehyde (63 mg, 0.45 mmol) in boiling methanol (5 ml) over 5 min and subsequently reacted with [Fe(py)_4_(NCS)_2_] (100 mg, 0.20 mmol) and ascorbic acid (11 mg, 0.06 mmol) in boiling methanol (5 ml). The formed yellow solution was slowly cooled to ambient temperature. Yellow–orange crystals then precipitated and were subsequently filtered off. Elemental analysis calculated (%) for C_21_H_32_FeN_10_S_2_: C, 46.32; H, 5.92; N, 25.72; S, 11.78. Found: C, 46.40; H, 6.10; N, 26.18; S, 11.80. IR *v* (cm^−1^, KBr): 1611 (C=N), 2071, 2116 (NCS).

## Refinement   

Crystal data, data collection and structure refinement details are summarized in Table 3 ▸. H atoms were positioned geometrically (C—H = 0.93–0.97 Å) and refined as riding with *U*_iso_(H) = 1.2*U*_eq_(C) or 1.5*U*_eq_(C-meth­yl).

## Supplementary Material

Crystal structure: contains datablock(s) I. DOI: 10.1107/S2056989021004412/dj2026sup1.cif

Structure factors: contains datablock(s) I. DOI: 10.1107/S2056989021004412/dj2026Isup2.hkl

Supporting information file. DOI: 10.1107/S2056989021004412/dj2026Isup3.cdx

The full colour-coded interaction mappings of a central reference molecule for the title compound. DOI: 10.1107/S2056989021004412/dj2026sup4.pdf

CCDC reference: 2079827

Additional supporting information:  crystallographic information; 3D view; checkCIF report

## Figures and Tables

**Table 1:** Hydrogen-bond geometry (Å, °)

*D*—H⋯*A*	*D*—H	H⋯*A*	*D*⋯*A*	*D*—H⋯*A*
C4—H4*B*⋯C21^i^	0.97	2.84	3.786 (4)	166
C5—H5⋯S1^ii^	0.93	2.99	3.718 (4)	137
C7—H7⋯S1^i^	0.93	2.90	3.764 (4)	155
C13—H13⋯S1^iii^	0.93	2.99	3.724 (4)	137
C13—H13⋯C20^iii^	0.93	2.75	3.558 (4)	146
C15—H15⋯S1^iii^	0.93	2.84	3.573 (4)	137
C17—H17*A*⋯S2^iv^	0.96	2.94	3.873 (4)	166
C17—H17*B*⋯S2^v^	0.96	2.94	3.850 (4)	158

Symmetry codes: (i) -x+1, -y+2, -z+1; (ii) x+1, y, z; (iii) -x, -y+1, -z+1; (iv) x, y-1, z; (v) -x, -y+1, -z.

**Table 2:** Comparison of the distortion parameters (Å, °) for the indicated Fe^II^ complexes

	<Fe—N>	*Σ*	*Θ*	CShM (*O*_h_)	CShM (*D*_3h_)
Title compound	2.170	127.8	438.2	3.829	6.709
IQEFAO	2.167	127.40	481.9	4.269	5.671
CUWQAP	2.186	149.38	453.2	6.285	4.008
CABLOH	1.899	178.16	725.74	12.735	0.525
BUNSAF	2.218	201.07	703.65	13.084	1.887
OWIHAE	2.202	206.57	894.48	16.909	0.602
OTANOO*^*a*^*	2.191	183.24	697.3	12.065	1.098

Note: (*a*) Parameters averaged over five independent complex cations.

**Table 3:** Experimental details

Crystal data	
Chemical formula	[Fe(NCS)_2_(C_19_H_32_N_8_)]
*M*_r_	544.53
Crystal system, space group	Triclinic, *P*\overline{1}
Temperature (K)	250
*a*, *b*, *c* (Å)	9.4768 (5), 10.8151 (5), 15.2493 (7)
α, β, γ (°)	102.267 (4), 102.813 (4), 103.291 (4)
*V* (Å^3^)	1424.90 (13)
*Z*	2
Radiation type	Mo *K*α
μ (mm^−1^)	0.70
Crystal size (mm)	0.4 × 0.2 × 0.2

Data collection	
Diffractometer	Rigaku Oxford Diffraction Xcalibur, Eos
Absorption correction	Multi-scan (*CrysAlis PRO*; Rigaku OD, 2015 ▸)
*T*_min_, *T*_max_	0.865, 1.000
No. of measured, independent and observed [*I* > 2σ(*I*)] reflections	10732, 5016, 4188
*R*_int_	0.025
(sin θ/λ)_max_ (Å^−1^)	0.595

Refinement	
*R*[*F* ^2^ > 2σ(*F* ^2^)], *wR*(*F* ^2^), *S*	0.038, 0.095, 1.04
No. of reflections	5016
No. of parameters	315
H-atom treatment	H-atom parameters constrained
Δρ_max_, Δρ_min_ (e Å^−3^)	0.46, −0.40

Computer programs: *CrysAlis PRO* (Rigaku OD, 2015 ▸), *SHELXT* (Sheldrick, 2015*a* ▸), *SHELXL2018/3* (Sheldrick, 2015*b* ▸) and *OLEX2* (Dolomanov *et al.*, 2009 ▸).

## supplementary crystallographic information

### Crystal data

**Table:**

[Fe(NCS)_2_(C_19_H_32_N_8_)]	*Z* = 2
*M**_r_* = 544.53	*F*(000) = 572
Triclinic, *P*1	*D*_x_ = 1.269 Mg m^−^^3^
*a* = 9.4768 (5) Å	Mo *K*α radiation, λ = 0.71073 Å
*b* = 10.8151 (5) Å	Cell parameters from 3729 reflections
*c* = 15.2493 (7) Å	θ = 2.0–26.8°
α = 102.267 (4)°	µ = 0.70 mm^−^^1^
β = 102.813 (4)°	*T* = 250 K
γ = 103.291 (4)°	Prism, orange
*V* = 1424.90 (13) Å^3^	0.4 × 0.2 × 0.2 mm

### Data collection

**Table:**

Rigaku Oxford Diffraction Xcalibur, Eos diffractometer	4188 reflections with *I* > 2σ(*I*)
Detector resolution: 16.1593 pixels mm^-1^	*R*_int_ = 0.025
ω scans	θ_max_ = 25.0°, θ_min_ = 2.0°
Absorption correction: multi-scan (CrysAlisPro; Rigaku OD, 2015)	*h* = −10→11
*T*_min_ = 0.865, *T*_max_ = 1.000	*k* = −12→12
10732 measured reflections	*l* = −18→18
5016 independent reflections	

### Refinement

**Table:**

Refinement on *F*^2^	Primary atom site location: dual
Least-squares matrix: full	Hydrogen site location: inferred from neighbouring sites
*R*[*F*^2^ > 2σ(*F*^2^)] = 0.038	H-atom parameters constrained
*wR*(*F*^2^) = 0.095	*w* = 1/[σ^2^(*F*_o_^2^) + (0.0371*P*)^2^ + 0.545*P*] where *P* = (*F*_o_^2^ + 2*F*_c_^2^)/3
*S* = 1.04	(Δ/σ)_max_ = 0.001
5016 reflections	Δρ_max_ = 0.46 e Å^−^^3^
315 parameters	Δρ_min_ = −0.40 e Å^−^^3^
0 restraints	

### Special details

**Table:**

Geometry. All esds (except the esd in the dihedral angle between two l.s. planes) are estimated using the full covariance matrix. The cell esds are taken into account individually in the estimation of esds in distances, angles and torsion angles; correlations between esds in cell parameters are only used when they are defined by crystal symmetry. An approximate (isotropic) treatment of cell esds is used for estimating esds involving l.s. planes.

### Fractional atomic coordinates and isotropic or equivalent isotropic displacement parameters (Å^2^)

**Table:**

	*x*	*y*	*z*	*U*_iso_*/*U*_eq_	
Fe1	0.23066 (4)	0.71954 (3)	0.39637 (2)	0.03412 (12)	
S1	−0.02770 (8)	0.91016 (6)	0.59945 (5)	0.04605 (18)	
S2	0.18981 (11)	0.99900 (8)	0.18833 (5)	0.0709 (3)	
N1	0.4279 (2)	0.81942 (18)	0.51741 (13)	0.0362 (5)	
N2	0.4378 (2)	0.70935 (19)	0.34658 (14)	0.0382 (5)	
N3	0.4618 (2)	0.6739 (2)	0.26556 (14)	0.0422 (5)	
N4	0.6061 (2)	0.73984 (19)	0.27554 (14)	0.0397 (5)	
N5	0.2174 (2)	0.57207 (18)	0.47997 (13)	0.0347 (4)	
N6	0.1170 (2)	0.52968 (18)	0.29454 (13)	0.0359 (5)	
N7	0.0625 (2)	0.48742 (19)	0.20347 (14)	0.0399 (5)	
N8	0.0220 (2)	0.35431 (18)	0.18153 (13)	0.0386 (5)	
N9	0.0762 (3)	0.7799 (2)	0.46068 (16)	0.0504 (6)	
N10	0.2124 (2)	0.8439 (2)	0.31141 (15)	0.0466 (5)	
C1	0.3934 (4)	0.7358 (3)	0.74168 (19)	0.0664 (9)	
H1A	0.488965	0.795885	0.780643	0.100*	
H1B	0.375591	0.655489	0.760243	0.100*	
H1C	0.314239	0.775546	0.748319	0.100*	
C2	0.5250 (3)	0.6455 (3)	0.6288 (2)	0.0570 (7)	
H2A	0.522605	0.621296	0.564042	0.086*	
H2B	0.513695	0.568366	0.651321	0.086*	
H2C	0.619922	0.709544	0.664560	0.086*	
C3	0.3958 (3)	0.7047 (2)	0.63914 (16)	0.0427 (6)	
C4	0.4194 (3)	0.8373 (2)	0.61365 (16)	0.0405 (6)	
H4A	0.336116	0.872821	0.620707	0.049*	
H4B	0.512065	0.900330	0.656164	0.049*	
C5	0.5577 (3)	0.8462 (2)	0.50359 (17)	0.0406 (6)	
H5	0.644384	0.894529	0.552199	0.049*	
C6	0.5647 (3)	0.7985 (2)	0.40914 (16)	0.0370 (6)	
C7	0.6733 (3)	0.8177 (2)	0.36351 (17)	0.0430 (6)	
H7	0.772226	0.872582	0.387956	0.052*	
C8	0.6700 (3)	0.7264 (3)	0.19420 (18)	0.0487 (7)	
C9	0.5645 (4)	0.6141 (4)	0.1149 (2)	0.1047 (15)	
H9A	0.550373	0.534196	0.134109	0.157*	
H9B	0.606340	0.604349	0.062876	0.157*	
H9C	0.468901	0.631363	0.096737	0.157*	
C10	0.6890 (6)	0.8558 (4)	0.1695 (3)	0.1240 (18)	
H10A	0.592928	0.873393	0.155227	0.186*	
H10B	0.727368	0.850811	0.116084	0.186*	
H10C	0.758785	0.925718	0.221622	0.186*	
C11	0.8222 (4)	0.7048 (5)	0.2252 (3)	0.1079 (15)	
H11A	0.887477	0.779295	0.275755	0.162*	
H11B	0.865439	0.695136	0.173773	0.162*	
H11C	0.810879	0.626152	0.245908	0.162*	
C12	0.2433 (3)	0.6048 (2)	0.58150 (16)	0.0436 (6)	
H12A	0.235390	0.523925	0.600421	0.052*	
H12B	0.163402	0.639851	0.596548	0.052*	
C13	0.1634 (3)	0.4524 (2)	0.43216 (17)	0.0388 (6)	
H13	0.155551	0.384125	0.460660	0.047*	
C14	0.1140 (3)	0.4256 (2)	0.33115 (16)	0.0343 (5)	
C15	0.0539 (3)	0.3127 (2)	0.25822 (17)	0.0416 (6)	
H15	0.038598	0.225792	0.261304	0.050*	
C16	−0.0388 (3)	0.2749 (3)	0.08089 (17)	0.0479 (7)	
C17	−0.1268 (4)	0.1368 (3)	0.0764 (2)	0.0696 (9)	
H17A	−0.060908	0.096972	0.110571	0.104*	
H17B	−0.166885	0.084533	0.012288	0.104*	
H17C	−0.208368	0.141301	0.103520	0.104*	
C18	0.0941 (4)	0.2720 (4)	0.0434 (2)	0.0891 (12)	
H18A	0.146877	0.360201	0.045494	0.134*	
H18B	0.059447	0.217558	−0.020213	0.134*	
H18C	0.161008	0.236346	0.080741	0.134*	
C19	−0.1432 (5)	0.3412 (3)	0.0304 (2)	0.0919 (13)	
H19A	−0.226092	0.341066	0.056965	0.138*	
H19B	−0.181430	0.293743	−0.034808	0.138*	
H19C	−0.088105	0.430716	0.037174	0.138*	
C20	0.0333 (3)	0.8349 (2)	0.51828 (18)	0.0367 (6)	
C21	0.2028 (3)	0.9064 (2)	0.25931 (17)	0.0390 (6)	

### Atomic displacement parameters (Å^2^)

**Table:**

	*U*^11^	*U*^22^	*U*^33^	*U*^12^	*U*^13^	*U*^23^
Fe1	0.0342 (2)	0.03043 (19)	0.0383 (2)	0.01036 (15)	0.01232 (16)	0.00759 (15)
S1	0.0547 (4)	0.0404 (4)	0.0486 (4)	0.0136 (3)	0.0264 (3)	0.0117 (3)
S2	0.0981 (7)	0.0621 (5)	0.0433 (4)	0.0113 (5)	0.0066 (4)	0.0230 (4)
N1	0.0387 (12)	0.0299 (10)	0.0377 (11)	0.0076 (9)	0.0129 (9)	0.0052 (9)
N2	0.0317 (11)	0.0383 (11)	0.0399 (12)	0.0052 (9)	0.0124 (9)	0.0040 (9)
N3	0.0318 (11)	0.0451 (12)	0.0424 (12)	0.0044 (9)	0.0111 (10)	0.0040 (10)
N4	0.0324 (11)	0.0432 (12)	0.0388 (12)	0.0054 (9)	0.0116 (9)	0.0059 (10)
N5	0.0328 (11)	0.0337 (11)	0.0369 (11)	0.0075 (9)	0.0115 (9)	0.0087 (9)
N6	0.0359 (11)	0.0324 (10)	0.0390 (12)	0.0085 (9)	0.0124 (9)	0.0090 (9)
N7	0.0447 (12)	0.0340 (11)	0.0402 (12)	0.0093 (9)	0.0125 (10)	0.0104 (9)
N8	0.0447 (12)	0.0324 (11)	0.0361 (11)	0.0068 (9)	0.0119 (9)	0.0082 (9)
N9	0.0538 (14)	0.0530 (14)	0.0625 (15)	0.0297 (12)	0.0297 (12)	0.0242 (12)
N10	0.0500 (14)	0.0437 (12)	0.0516 (14)	0.0159 (11)	0.0188 (11)	0.0181 (11)
C1	0.079 (2)	0.072 (2)	0.0385 (16)	0.0090 (17)	0.0176 (15)	0.0089 (15)
C2	0.0541 (18)	0.0564 (18)	0.0582 (18)	0.0216 (14)	0.0080 (15)	0.0133 (15)
C3	0.0466 (15)	0.0469 (15)	0.0328 (13)	0.0131 (12)	0.0118 (12)	0.0075 (11)
C4	0.0392 (14)	0.0411 (14)	0.0360 (13)	0.0102 (11)	0.0125 (11)	−0.0006 (11)
C5	0.0345 (14)	0.0369 (13)	0.0413 (14)	0.0027 (11)	0.0086 (11)	0.0031 (11)
C6	0.0312 (13)	0.0359 (13)	0.0377 (13)	0.0042 (10)	0.0091 (11)	0.0046 (11)
C7	0.0322 (14)	0.0458 (15)	0.0391 (14)	−0.0006 (11)	0.0081 (11)	0.0022 (12)
C8	0.0420 (15)	0.0608 (17)	0.0390 (14)	0.0065 (13)	0.0173 (12)	0.0078 (13)
C9	0.079 (3)	0.138 (4)	0.056 (2)	−0.011 (2)	0.0318 (19)	−0.025 (2)
C10	0.209 (6)	0.103 (3)	0.096 (3)	0.043 (3)	0.095 (4)	0.051 (3)
C11	0.063 (2)	0.195 (5)	0.072 (2)	0.055 (3)	0.034 (2)	0.014 (3)
C12	0.0479 (15)	0.0437 (15)	0.0394 (14)	0.0078 (12)	0.0188 (12)	0.0114 (12)
C13	0.0397 (14)	0.0334 (13)	0.0429 (14)	0.0056 (11)	0.0123 (11)	0.0147 (11)
C14	0.0328 (13)	0.0313 (12)	0.0388 (13)	0.0076 (10)	0.0121 (11)	0.0096 (10)
C15	0.0528 (16)	0.0313 (13)	0.0401 (14)	0.0090 (12)	0.0134 (12)	0.0121 (11)
C16	0.0617 (18)	0.0425 (15)	0.0342 (14)	0.0104 (13)	0.0118 (13)	0.0069 (12)
C17	0.094 (3)	0.0498 (17)	0.0418 (16)	−0.0047 (17)	0.0119 (16)	0.0000 (14)
C18	0.088 (3)	0.103 (3)	0.059 (2)	0.004 (2)	0.039 (2)	−0.006 (2)
C19	0.117 (3)	0.079 (2)	0.057 (2)	0.032 (2)	−0.017 (2)	0.0112 (18)
C20	0.0338 (13)	0.0346 (13)	0.0487 (15)	0.0130 (11)	0.0141 (12)	0.0202 (12)
C21	0.0364 (14)	0.0363 (13)	0.0354 (14)	0.0057 (11)	0.0075 (11)	−0.0003 (12)

### Geometric parameters (Å, º)

**Table:**

Fe1—N1	2.182 (2)	C4—H4B	0.9700
Fe1—N2	2.2733 (19)	C5—H5	0.9300
Fe1—N5	2.2422 (19)	C5—C6	1.445 (3)
Fe1—N6	2.1619 (19)	C6—C7	1.367 (3)
Fe1—N9	2.082 (2)	C7—H7	0.9300
Fe1—N10	2.066 (2)	C8—C9	1.489 (4)
S1—C20	1.623 (3)	C8—C10	1.507 (4)
S2—C21	1.628 (3)	C8—C11	1.502 (4)
N1—C4	1.462 (3)	C9—H9A	0.9600
N1—C5	1.273 (3)	C9—H9B	0.9600
N2—N3	1.300 (3)	C9—H9C	0.9600
N2—C6	1.361 (3)	C10—H10A	0.9600
N3—N4	1.347 (3)	C10—H10B	0.9600
N4—C7	1.345 (3)	C10—H10C	0.9600
N4—C8	1.491 (3)	C11—H11A	0.9600
N5—C12	1.463 (3)	C11—H11B	0.9600
N5—C13	1.264 (3)	C11—H11C	0.9600
N6—N7	1.307 (3)	C12—H12A	0.9700
N6—C14	1.356 (3)	C12—H12B	0.9700
N7—N8	1.348 (3)	C13—H13	0.9300
N8—C15	1.338 (3)	C13—C14	1.451 (3)
N8—C16	1.498 (3)	C14—C15	1.370 (3)
N9—C20	1.156 (3)	C15—H15	0.9300
N10—C21	1.149 (3)	C16—C17	1.513 (4)
C1—H1A	0.9600	C16—C18	1.498 (4)
C1—H1B	0.9600	C16—C19	1.519 (4)
C1—H1C	0.9600	C17—H17A	0.9600
C1—C3	1.535 (3)	C17—H17B	0.9600
C2—H2A	0.9600	C17—H17C	0.9600
C2—H2B	0.9600	C18—H18A	0.9600
C2—H2C	0.9600	C18—H18B	0.9600
C2—C3	1.529 (4)	C18—H18C	0.9600
C3—C4	1.545 (3)	C19—H19A	0.9600
C3—C12	1.531 (3)	C19—H19B	0.9600
C4—H4A	0.9700	C19—H19C	0.9600
			
N1—Fe1—N2	73.16 (7)	N4—C7—H7	127.5
N1—Fe1—N5	78.59 (7)	C6—C7—H7	127.5
N5—Fe1—N2	102.76 (7)	N4—C8—C10	106.5 (2)
N6—Fe1—N1	141.68 (7)	N4—C8—C11	107.9 (2)
N6—Fe1—N2	86.29 (7)	C9—C8—N4	109.4 (2)
N6—Fe1—N5	74.84 (7)	C9—C8—C10	111.6 (3)
N9—Fe1—N1	95.38 (8)	C9—C8—C11	111.7 (3)
N9—Fe1—N2	164.79 (8)	C11—C8—C10	109.5 (3)
N9—Fe1—N5	84.21 (8)	C8—C9—H9A	109.5
N9—Fe1—N6	108.70 (8)	C8—C9—H9B	109.5
N10—Fe1—N1	108.35 (8)	C8—C9—H9C	109.5
N10—Fe1—N2	82.59 (8)	H9A—C9—H9B	109.5
N10—Fe1—N5	172.39 (8)	H9A—C9—H9C	109.5
N10—Fe1—N6	100.36 (8)	H9B—C9—H9C	109.5
N10—Fe1—N9	91.91 (8)	C8—C10—H10A	109.5
C4—N1—Fe1	122.55 (15)	C8—C10—H10B	109.5
C5—N1—Fe1	118.20 (16)	C8—C10—H10C	109.5
C5—N1—C4	118.5 (2)	H10A—C10—H10B	109.5
N3—N2—Fe1	135.06 (16)	H10A—C10—H10C	109.5
N3—N2—C6	110.07 (19)	H10B—C10—H10C	109.5
C6—N2—Fe1	110.64 (15)	C8—C11—H11A	109.5
N2—N3—N4	106.56 (18)	C8—C11—H11B	109.5
N3—N4—C8	120.84 (19)	C8—C11—H11C	109.5
C7—N4—N3	110.97 (19)	H11A—C11—H11B	109.5
C7—N4—C8	128.1 (2)	H11A—C11—H11C	109.5
C12—N5—Fe1	124.97 (15)	H11B—C11—H11C	109.5
C13—N5—Fe1	115.10 (16)	N5—C12—C3	115.17 (19)
C13—N5—C12	119.2 (2)	N5—C12—H12A	108.5
N7—N6—Fe1	135.88 (15)	N5—C12—H12B	108.5
N7—N6—C14	109.98 (18)	C3—C12—H12A	108.5
C14—N6—Fe1	113.70 (15)	C3—C12—H12B	108.5
N6—N7—N8	106.35 (18)	H12A—C12—H12B	107.5
N7—N8—C16	119.69 (19)	N5—C13—H13	121.3
C15—N8—N7	111.10 (19)	N5—C13—C14	117.4 (2)
C15—N8—C16	129.1 (2)	C14—C13—H13	121.3
C20—N9—Fe1	158.0 (2)	N6—C14—C13	118.2 (2)
C21—N10—Fe1	175.3 (2)	N6—C14—C15	107.5 (2)
H1A—C1—H1B	109.5	C15—C14—C13	134.2 (2)
H1A—C1—H1C	109.5	N8—C15—C14	105.1 (2)
H1B—C1—H1C	109.5	N8—C15—H15	127.5
C3—C1—H1A	109.5	C14—C15—H15	127.5
C3—C1—H1B	109.5	N8—C16—C17	108.3 (2)
C3—C1—H1C	109.5	N8—C16—C19	107.8 (2)
H2A—C2—H2B	109.5	C17—C16—C19	110.0 (3)
H2A—C2—H2C	109.5	C18—C16—N8	107.2 (2)
H2B—C2—H2C	109.5	C18—C16—C17	110.8 (3)
C3—C2—H2A	109.5	C18—C16—C19	112.5 (3)
C3—C2—H2B	109.5	C16—C17—H17A	109.5
C3—C2—H2C	109.5	C16—C17—H17B	109.5
C1—C3—C4	106.4 (2)	C16—C17—H17C	109.5
C2—C3—C1	109.7 (2)	H17A—C17—H17B	109.5
C2—C3—C4	111.0 (2)	H17A—C17—H17C	109.5
C2—C3—C12	110.4 (2)	H17B—C17—H17C	109.5
C12—C3—C1	106.8 (2)	C16—C18—H18A	109.5
C12—C3—C4	112.4 (2)	C16—C18—H18B	109.5
N1—C4—C3	110.89 (19)	C16—C18—H18C	109.5
N1—C4—H4A	109.5	H18A—C18—H18B	109.5
N1—C4—H4B	109.5	H18A—C18—H18C	109.5
C3—C4—H4A	109.5	H18B—C18—H18C	109.5
C3—C4—H4B	109.5	C16—C19—H19A	109.5
H4A—C4—H4B	108.1	C16—C19—H19B	109.5
N1—C5—H5	121.4	C16—C19—H19C	109.5
N1—C5—C6	117.1 (2)	H19A—C19—H19B	109.5
C6—C5—H5	121.4	H19A—C19—H19C	109.5
N2—C6—C5	117.0 (2)	H19B—C19—H19C	109.5
N2—C6—C7	107.4 (2)	N9—C20—S1	179.2 (2)
C7—C6—C5	135.5 (2)	N10—C21—S2	178.1 (2)
N4—C7—C6	105.0 (2)		
			
Fe1—N1—C4—C3	72.0 (2)	N7—N6—C14—C15	0.0 (3)
Fe1—N1—C5—C6	−4.9 (3)	N7—N8—C15—C14	1.3 (3)
Fe1—N2—N3—N4	154.24 (17)	N7—N8—C16—C17	−159.3 (2)
Fe1—N2—C6—C5	20.5 (3)	N7—N8—C16—C18	81.1 (3)
Fe1—N2—C6—C7	−161.09 (17)	N7—N8—C16—C19	−40.3 (3)
Fe1—N5—C12—C3	−55.6 (3)	C1—C3—C4—N1	178.3 (2)
Fe1—N5—C13—C14	−1.4 (3)	C1—C3—C12—N5	174.3 (2)
Fe1—N6—N7—N8	172.44 (16)	C2—C3—C4—N1	59.1 (3)
Fe1—N6—C14—C13	9.3 (3)	C2—C3—C12—N5	−66.5 (3)
Fe1—N6—C14—C15	−173.69 (16)	C4—N1—C5—C6	165.5 (2)
N1—C5—C6—N2	−11.6 (3)	C4—C3—C12—N5	58.0 (3)
N1—C5—C6—C7	170.7 (3)	C5—N1—C4—C3	−97.9 (3)
N2—N3—N4—C7	−0.1 (3)	C5—C6—C7—N4	178.3 (3)
N2—N3—N4—C8	−177.5 (2)	C6—N2—N3—N4	0.4 (3)
N2—C6—C7—N4	0.4 (3)	C7—N4—C8—C9	171.2 (3)
N3—N2—C6—C5	−178.9 (2)	C7—N4—C8—C10	−68.0 (4)
N3—N2—C6—C7	−0.5 (3)	C7—N4—C8—C11	49.5 (4)
N3—N4—C7—C6	−0.2 (3)	C8—N4—C7—C6	177.0 (2)
N3—N4—C8—C9	−11.8 (4)	C12—N5—C13—C14	169.1 (2)
N3—N4—C8—C10	108.9 (3)	C12—C3—C4—N1	−65.1 (3)
N3—N4—C8—C11	−133.5 (3)	C13—N5—C12—C3	134.8 (2)
N5—C13—C14—N6	−5.3 (3)	C13—C14—C15—N8	175.5 (3)
N5—C13—C14—C15	178.7 (3)	C14—N6—N7—N8	0.8 (2)
N6—N7—N8—C15	−1.3 (3)	C15—N8—C16—C17	25.7 (4)
N6—N7—N8—C16	−177.2 (2)	C15—N8—C16—C18	−94.0 (3)
N6—C14—C15—N8	−0.7 (3)	C15—N8—C16—C19	144.7 (3)
N7—N6—C14—C13	−177.0 (2)	C16—N8—C15—C14	176.7 (2)

### Hydrogen-bond geometry (Å, º)

**Table:**

*D*—H···*A*	*D*—H	H···*A*	*D*···*A*	*D*—H···*A*
C4—H4*B*···C21^i^	0.97	2.84	3.786 (4)	166
C5—H5···S1^ii^	0.93	2.99	3.718 (4)	137
C7—H7···S1^i^	0.93	2.90	3.764 (4)	155
C13—H13···S1^iii^	0.93	2.99	3.724 (4)	137
C13—H13···C20^iii^	0.93	2.75	3.558 (4)	146
C15—H15···S1^iii^	0.93	2.84	3.573 (4)	137
C17—H17*A*···S2^iv^	0.96	2.94	3.873 (4)	166
C17—H17*B*···S2^v^	0.96	2.94	3.850 (4)	158

Symmetry codes: (i) −*x*+1, −*y*+2, −*z*+1; (ii) *x*+1, *y*, *z*; (iii) −*x*, −*y*+1, −*z*+1; (iv) *x*, *y*−1, *z*; (v) −*x*, −*y*+1, −*z*.
