# Supplementary material for: Crystal structure of {N 1,N 3-bis­[(1-tert-butyl-1H-1,2,3-triazol-4-yl)methyl­idene]-2,2-di­methyl­propane-1,3-di­amine}­bis­(thio­cyanato)­iron(II)
Source: Acta Crystallogr E Crystallogr Commun. 2021 Apr 30;77(Pt 5):573–8. doi: 10.1107/S2056989021004412 (PMC8100266; doi:10.1107/S2056989021004412)
Supplement: Supplementary file 4 [file e-77-00573-sup4.pdf]

Supplementary file S1

The full colour-coded interaction mappings of a central reference molecule for the title compound.

Full details of the various contributions to the total energy (E_{tot}) are also given.

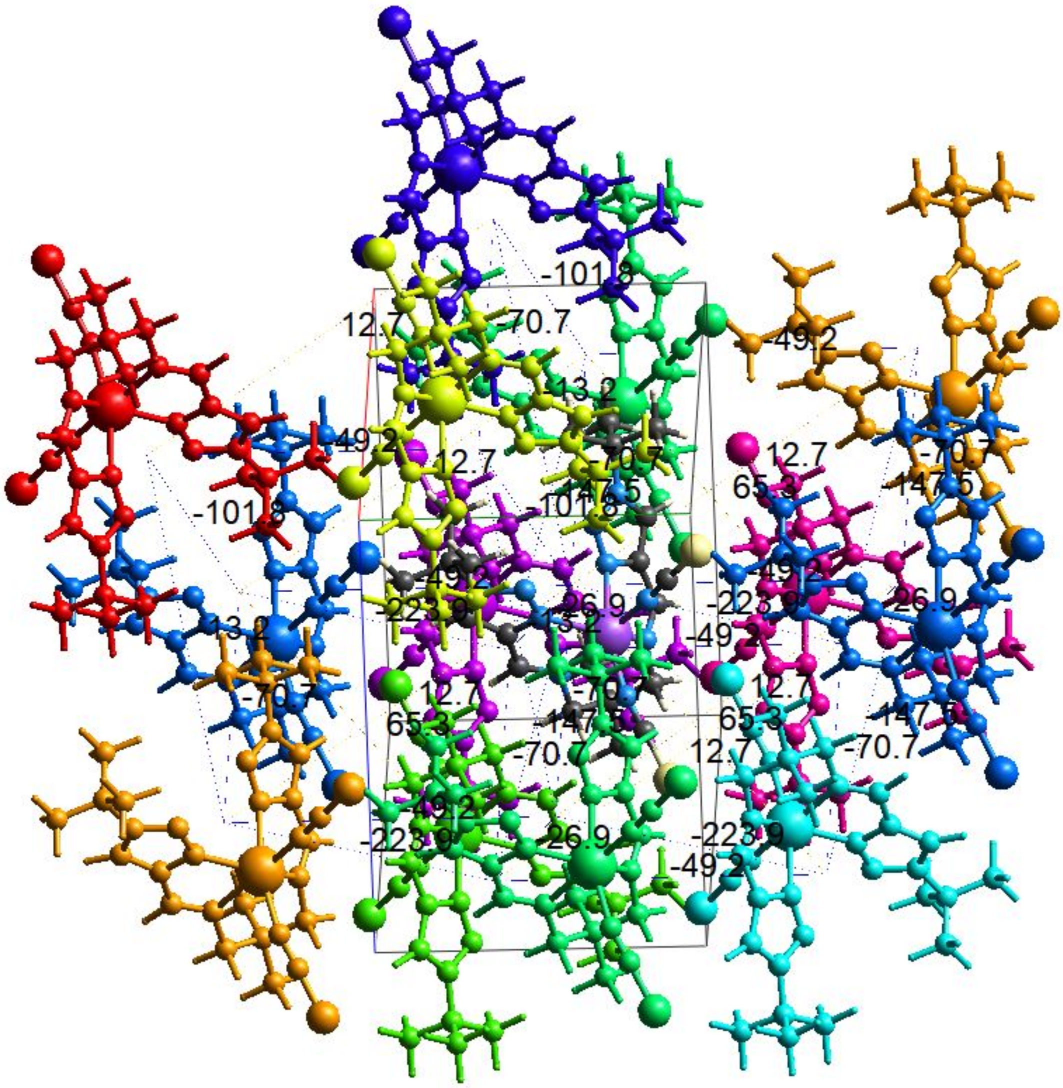

	N	Sym_op	R	Electron Density	E_ele	E_pol	E_dis	E_rep	E_tot
	1	-x, -y, -z	15.06	B3LYP/6-31G(d,p)	9.2	-1.5	-5.7	0.5	4.0
	1	x, y, z	12.64	B3LYP/6-31G(d,p)	18.6	-2.5	-7.0	1.6	12.7
	1	-x, -y, -z	10.24	B3LYP/6-31G(d,p)	-69.9	-28.2	-37.6	41.8	-101.8
	1	-x, -y, -z	8.37	B3LYP/6-31G(d,p)	-104.8	-37.1	-68.0	80.8	-147.5
	1	x, y, z	9.48	B3LYP/6-31G(d,p)	-41.1	-19.6	-36.5	30.7	-70.7
	1	-x, -y, -z	10.59	B3LYP/6-31G(d,p)	74.3	-17.8	-20.7	28.9	65.3
	1	x, y, z	10.82	B3LYP/6-31G(d,p)	-39.0	-11.2	-10.7	15.7	-49.2
	1	-x, -y, -z	12.54	B3LYP/6-31G(d,p)	2.2	-3.1	-21.5	8.9	-13.2
	1	-x, -y, -z	7.65	B3LYP/6-31G(d,p)	15.1	-5.4	-59.2	20.5	-26.9
	1	-x, -y, -z	7.30	B3LYP/6-31G(d,p)	-157.6	-62.5	-84.8	101.7	-223.9
